# Development, Optimization, and Evaluation of New Gel Formulations with Cyclodextrin Complexes and Volatile Oils with Antimicrobial Activity

**DOI:** 10.3390/gels10100645

**Published:** 2024-10-10

**Authors:** Alina Ionela Stancu, Eliza Oprea, Lia Mara Dițu, Anton Ficai, Cornelia-Ioana Ilie, Irinel Adriana Badea, Mihaela Buleandra, Oana Brîncoveanu, Mihaela Violeta Ghica, Ionela Avram, Cristina Elena Dinu Pîrvu, Magdalena Mititelu

**Affiliations:** 1 Department Science and Engineering of Oxide Materials and Nanomaterials, Faculty of Chemical Engineering and Biotechnology, National University of Science and Technology Politehnica Bucharest, 1–7 Polizu Street, 011061 Bucharest, Romania; alina.stancu1995@gmail.com (A.I.S.);; 2 Department of Botany and Microbiology, Faculty of Biology, University of Bucharest, Portocalilor 1-3, 060101 Bucharest, Romania; lia-mara.ditu@bio.unibuc.ro; 3 MICROGEN Research Centre, Faculty of Biology, University of Bucharest, Portocalilor 1-3, 060101 Bucharest, Romania; ionela.sarbu@bio.unibuc.ro; 4 Academy of Romanian Scientists, Ilfov Street 1-3, 050045 Bucharest, Romania; 5 National Centre for Micro and Nanomaterials and National Centre for Food Safety, National University of Science and Technology Politehnica Bucharest, 060042 Bucharest, Romania; 6 Department of Analytical Chemistry and Physical Chemistry, Faculty of Chemistry, University of Bucharest, 90–92 Panduri Street, 050663 Bucharest, Romania; irinel.badea@chimie.unibuc.ro (I.A.B.); mihaela.buleandra@g.unibuc.ro (M.B.); 7 National Institute for Research and Development in Microtechnologies, 126A Erou Iancu Nicolae Street, 077190 Bucharest, Romania; oana.brincoveanu@imt.ro; 8 Department of Physical and Colloidal Chemistry, Faculty of Pharmacy, University of Medicine and Pharmacy Carol Davila, 6 Traian Vuia Street, 020956 Bucharest, Romania; mihaela.ghica@umfcd.ro (M.V.G.); cristina.dinu@umfcd.ro (C.E.D.P.); 9 Innovative Therapeutic Structures Research and Development Centre (InnoTher), University of Medicine and Pharmacy Carol Davila, 6 Traian Vuia Street, 020956 Bucharest, Romania; 10 Department of Genetics, Faculty of Biology, University of Bucharest, Portocalilor 1-3, 060101 Bucharest, Romania; 11 Department of Clinical Laboratory and Food Safety, Faculty of Pharmacy, University of Medicine and Pharmacy Carol Davila, 6 Traian Vuia Street, 020956 Bucharest, Romania; magdalena.mititelu@umfcd.ro

**Keywords:** antimicrobial agents, β-cyclodextrin complex, volatile oils, hydrogels

## Abstract

This study aimed to develop and evaluate hydrogels containing a cyclodextrin complex with clove essential oil and other free volatile oils with antimicrobial properties (tea tree and rosemary essential oils), focusing on their pharmaco-technical and rheological characteristics. The formulations varied in the Carbopol 940 (a hydrophilic polymer) and volatile oils’ concentrations. Rheological analysis indicated that the gels displayed pseudoplastic behavior, with the flow index (n) values below 1, ensuring appropriate consistency and handling. The results showed that increasing the Carbopol concentration significantly enhanced the yield stress, consistency index, and viscosity, with gel B, containing 1% Carbopol, 1.5% tea tree essential oil, and 1.5% rosemary essential oil, demonstrating optimal stability and rheological properties. At the same time, the concentration of volatile oils was found to modulate the gels’ flow parameters, but their effect was less pronounced than that of the gel-forming polymer. Antimicrobial testing revealed that both gel B and gel E (containing 1% Carbopol, 2% tea tree essential oil, and 2% rosemary essential oil) exhibited antimicrobial activity against Gram-positive, Gram-negative bacteria, and *Candida* spp., with gel E showing superior efficacy against *Candida tropicalis*. The antimicrobial effects were likely influenced by the higher concentrations of tea tree and rosemary essential oils in gel E. Overall, the study demonstrates that the concentration of Carbopol 940 primarily determines the gel’s rheological behavior, while volatile oil concentration modulates antimicrobial effectiveness.

## 1. Introduction

Hydrogels are aqueous or hydro-glycerinated gels in which macromolecules form a three-dimensional network comprising the entire liquid phase. The active substances could be easily released if not integrated into or bound to this polymer network. The pores in the gel network allow relatively free diffusion of molecules of active substances with not too large dimensions. Consistency is chosen depending on the mode of administration, the climatic conditions endured by the preparations, and the nature of the active principles, which can change the viscosity of the gel base, acting on the polymer bonds [1,2,3]. The growing demand for developing new hydrogels is mainly due to their wide range of applications, namely hygiene and personal care products, pharmaceuticals and cosmetics, food and agriculture, and more recently, tissue engineering or regenerative medicine. The use of hydrogels is based on the following advantages: they are washable, are preferred for people with oily skin, for the treatment of eye diseases and the treatment of hairy regions or mucous membranes, have good skin tolerance, the drug absorptions from these bases are superior to fatty bases, they are compatible with many active substances, however, taking into account the ionic character of both the ointment bases and the incorporated active substances [4,5,6]. Nevertheless, some disadvantages can be mentioned, such as rapid water loss and susceptibility to microbial invasion, which requires the mandatory addition of preservatives.

Antimicrobial hydrogels and essential oils in topical formulations have been widely studied, but challenges such as stability, bioavailability, and efficacy in long-term use need more studies. Recent studies have explored the development of advanced antimicrobial hydrogels, yet issues with the stability and effectiveness of essential oils, particularly their rapid degradation or volatility, limit their potential for sustained therapeutic effects. For instance, according to recent findings, hydrogels formulated with cyclodextrin complexes demonstrate enhanced stability and controlled release of bioactive agents, making them promising for biomedical applications. Specifically, carbopol-based hydrogels with cyclodextrin complexes have shown improved drug delivery performance due to their capacity to form inclusion complexes with diverse compounds, such as eugenol [7], thymol [8], clotrimazole [9], etc.

Additionally, a recent study highlighted essential oils’ role in enhancing hydrogel antimicrobial properties. Still, these oils are prone to degradation and require encapsulation or stabilization to prolong their activity [10].

Furthermore, cyclodextrins based hydrogels have gained special attention due to their properties (extended polymeric network, dynamic stiffness like an extracellular matrix, biocompatibility, etc.) and their potential for biomedical applications [11]. Moreover, adding essential oils with antimicrobial properties in such formulations enhances their wound-healing properties, which could be more potent than conventional antimicrobial drugs [12,13].

Hydrophilic gels are obtained from colloidal macromolecules, which have the property of soaking with water or other hydrophilic solvents by absorption. Carbopol is an active acrylic acid polymer anionic that forms a low-density white powder and gels in concentrations ranging from 0.5 to 1.5% [14].

Carbopol can be used to process non-greasy ointments, is easily washable, stable, non-irritating to the skin, and has rapid release. Several varieties of Carbopol are known: 940, 974, and ULTREZ 10. To obtain the hydrogel, the Carbopol is dispersed under stirring in a preservative solution, left to hydrate, and then neutralized with a base (sodium, potassium, triethanolamine hydroxides), when the acid polymer passes into a soluble state. This is the mixed slowly and completed with preservative solution. Formulation characteristics, including viscosity, elasticity, and rheology, are the most important factors in semisolid preparation development and the final behavior of semisolid preparations [15].

One of the major advantages of topical administration for the local action of a drug substance is the avoidance of side effects at the systemic level. Macromolecular agents forming semisolid structures of the carbomer type are polymers of acrylic acid, depending on the sort of different molecular weights and degrees of crosslinking. The assigned role is usually correlated with local skin or mucous applications. The emulsifier’s role is mentioned less and is associated with low concentration levels (0.1–0.5%). This aspect was considered when selecting the composition of emulsions containing volatile oils. Outstanding biocompatibility is doubled by adequate microstructure control through quality attributes such as pH value or neutralizing agent used (hydroxide alkaline or ethanolamine derivative) [16,17].

This study focused on the development and comprehensive evaluation of hydrogels formulated with a cyclodextrin complex containing clove essential oil (CO), as well as hydrogels incorporating free volatile oils such as tea tree and rosemary, known for their antimicrobial properties. The research aimed to assess the pharmaco-technical properties of these hydrogels, including their stability, texture, ease of application, and rheological behavior, which is crucial for understanding the flow and deformation characteristics of the gels. By combining these natural antimicrobial agents with cyclodextrin, the study sought to enhance the stability and effectiveness of the volatile oils, leading to innovative topical formulations with improved therapeutic potential.

## 2. Results and Discussion

### 2.1. GC-MS Analysis

The chemical composition of *Melaleuca alternifolia* (tea tree) essential oil by GC-MS is presented in Table 1.

The chemical composition of *Rosmarinus officinalis* (rosemary) essential oil by GC-MS is shown in Table 2.

GC-MS analysis showed that the most abundant constituent of the *Melaleuca alternifolia* volatile oil is terpinen-4-ol (36.32%), followed by γ-terpinene (18.85%) and α-terpinene (10.85%). A study from 2019 [18] confirmed that these three components are the major constituents of the tea tree essential oil (TTO), taking into account small variations of percentages.

For *Rosmarinus officinalis*, the most abundant component of the volatile oil (RO) is eucalyptol (49.70%), followed by camphor (14.12%), α-pinene (11.83%), and β-caryophyllene (5.80%). The literature data confirm the results obtained. Studies [19,20] conducted on volatile oil hydrodistilled from different parts of *Rosmarinus officinalis* L. also showed that the major component of the oil is eucalyptol, followed by α-pinene and camphor. Furthermore, significant amounts of borneol, camphene, and verbenone were found.

### 2.2. Gels Characteristics

Carbopol is a widely used polymer in hydrogel formulations due to its excellent gelling properties, ease of use, and biocompatibility. The 0.5–1.5% concentration range is commonly employed in pharmaceutical and cosmetic formulations to achieve optimal gel viscosity, texture, and stability. In particular, a concentration of 1% is often chosen as it offers a balance between effective gel formation and ease of application without being overly dense or challenging to spread. The literature frequently cites 1% Carbopol as a standard concentration that forms a stable and easily applicable hydrogel. Studies have demonstrated that at this concentration, Carbopol provides sufficient viscosity and structure to support the incorporation of active substances while maintaining a smooth texture suitable for topical applications [21]. Higher concentrations, such as 2%, are sometimes used when a firmer gel consistency is required or when higher viscosities are necessary for a specific delivery route (e.g., localized skin application). However, excessively high concentrations can result in too thick or difficult to spread gels, thus limiting patient compliance [22]. In addition, volatile oils such as tea tree, rosemary, and clove are known for their antimicrobial properties and are often used in topical formulations. The concentration of these oils in a formulation is critical to balancing efficacy with potential skin irritation. A concentration of 2% essential oil is commonly employed in topical formulations, providing potent antimicrobial activity while minimizing the irritation risk. According to studies on essential oil-based hydrogels, concentrations in the range of 1–3% are optimal for achieving therapeutic benefits without overwhelming the formulation with too much oil, which could affect the stability or texture of the gel [23]. Additionally, studies on wound healing formulations often recommend using essential oils at concentrations around 2% to ensure both efficacy and patient comfort [24]. Furthermore, combining different volatile oils at this concentration generates synergistic antimicrobial effects without increasing the risk of adverse reactions or compromising the overall gel structure.

Preliminary experiments involved testing various Carbopol and essential oils concentrations to determine the optimal formulation. These tests have guided the decision to use 1% Carbopol and 2% essential oils, as they produced hydrogels with suitable rheological properties, ease of application, and antimicrobial potential.

TTO is well known for its potent antimicrobial and antifungal properties and has been extensively studied for its biocompatibility in topical applications. Many studies show it is safe for humans when used in concentrations up to 5%. The TTO used in this study at 2% concentration aligns with established safe concentrations, allowing the oil to exert its antimicrobial effects without posing significant toxicity risks [25].

RO contains compounds like carnosic acid and rosmarinic acid, which are known for their antioxidant, antimicrobial, and anti-inflammatory properties. It has been used in various cosmetic and medicinal formulations and is considered biocompatible for topical applications. Studies have demonstrated that RO is well tolerated by the skin at concentrations typically used in therapeutic products (1–2%). It has also been shown to enhance wound healing and reduce skin irritation, making it a suitable choice for incorporation into hydrogels [26].

The TTO, CO, and RO selection considered the synergistic antimicrobial activity, which can enhance the overall therapeutic outcome while allowing for lower concentrations of each oil. This reduces the likelihood of adverse reactions associated with higher concentrations of a single oil. At the same time, combining these essential oils leverages their complementary properties: analgesic and anti-inflammatory benefits (CO), broad-spectrum antimicrobial activity (TTO), and wound healing and antioxidant protection (RO). The combined use of these oils ensures a balanced formulation that maximizes therapeutic benefits while minimizing toxicity risks [27].

Gels containing RO loaded into lipid nanoparticles (containing 3% RO *w*/*w*) increased skin hydration and elasticity compared to gels containing free RO in human volunteers [28].

Regarding biocompatibility, eugenol is known to promote wound healing and has been used historically in dental care due to its ability to reduce pain and inflammation. This main active compound from CO has been shown to have good biocompatibility when used in appropriate concentrations for topical applications. Research has demonstrated that low concentrations of CO (1–3%) in topical formulations are generally safe and well tolerated, although higher concentrations may cause skin irritation [29]. Therefore, the study uses CO at controlled concentrations to ensure formula efficacy and safety.

RO is considered non-toxic when used in topical formulations at concentrations typically below 5%. Higher concentrations can irritate, but its incorporation at 2% in the study’s hydrogel formulation is consistent with safety recommendations. Rosemary essential oil (RO) is also less likely to cause allergic reactions than other essential oils, enhancing its suitability for sensitive or damaged skin [28]. In addition, gels containing RO loaded into lipid nanoparticles (containing 3% RO *w*/*w*) increased skin hydration and elasticity compared to gels containing free RO in human volunteers [28]. This study suggested that gel formulations are indicated for treating various surface cutaneous alterations.

TTO is generally considered safe for topical use in 1–5% concentrations, but it can be toxic if ingested or applied at very high concentrations. Prolonged exposure to high concentrations of TTO can lead to allergic reactions or skin sensitization in some individuals. The concentration chosen in this study (2%) is below the toxicity threshold and aligns with the safe use guidelines for maintaining efficacy without causing adverse effects [30]. Moreover, the chitosan/polyvinyl alcohol films containing 1.0 and 1.5% of TTO showed signs of reabsorption comparable to those of porcine collagen, indicating that the oil’s incorporation promotes more significant reabsorption and biocompatibility, stimulating a more substantial interaction with phagocytic cells [31].

Considering that it is essential to know aspects related to toxicity in order to develop a pharmaceutical product, TTO, RO, and CO were chosen as bioactive components of the formulations for their low toxicity and antimicrobial activity against various bacterial strains. These two properties being supported by both the literature data and the results presented further in this study.

Thus, studies conducted on Winstar rats demonstrate that the LD_50_ is greater than 2.000 mg/kg of body weight in the case of RO and between 1.9–2.6 mL/kg in the case of TTO when administrated orally [30,32], while CO toxicity against albino rats led to a LD_50_ value of 3597.5 mg/kg [33].

The characteristics of the gel samples are presented in Table 3 and Table 4.

The data from Table 3 and Table 4 show that the gels are homogenous. All gels presented good stability by maintaining a homogenous appearance at 2 °C and 40 °C, even after 30 days. A concentration in volatile oils over 8% produces an un-homogenous appearance, and the formulas become unstable.

**Figure 1:**
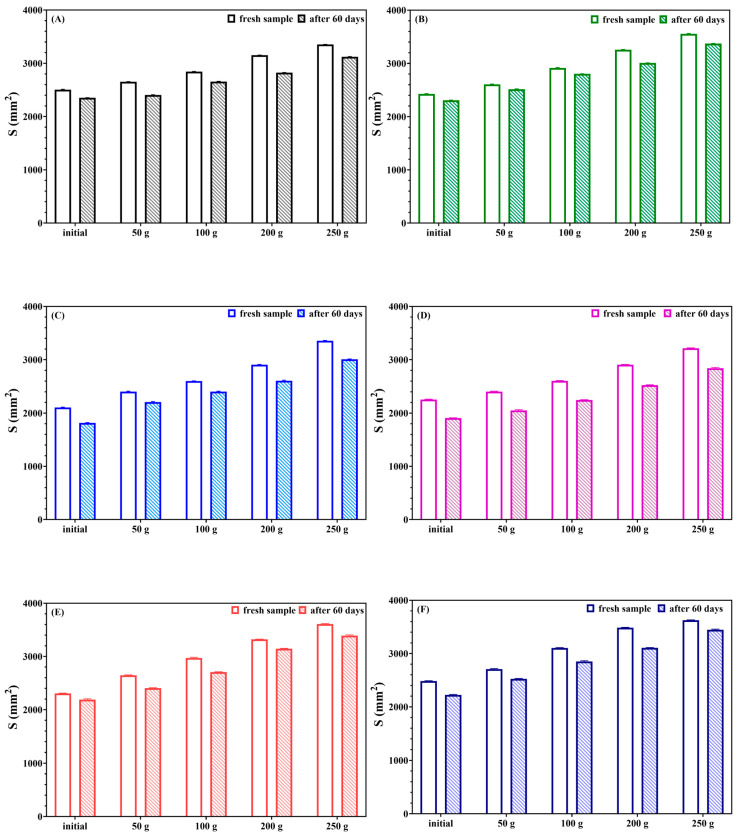
Spreading area function of the applied mass for samples. (**A**–**F**) represent the gel formulas A, B, C, D, E, and F. The differences between samples (fresh vs. 60 days) for each formula were statistically analyzed using one-way ANOVA and Tukey’s multiple comparisons test. The resulting data were statistically significant (*p* < 0.05).

As observed in Figure 1, the analysis of the spreading area of the gels revealed significant variations attributable to differences in their consistency. The experimental data demonstrated that all the prepared formulations were stable and homogeneous, displaying good consistency and a spreading capacity that remained within desirable limits over time. Notably, formulations B, E, and F exhibited the highest values for spreading area, indicating superior spread ability, which is an important characteristic for topical applications. The stability and rheological studies further highlighted the robustness and optimal rheological properties of formula B. Specifically, formula B demonstrated excellent stability, maintaining its structural integrity and performance over time. Its optimal rheological characteristics, such as viscosity and flow behavior, make it particularly suitable for applications where consistent texture and ease of application are essential. Given these promising results, the concentration of carbopol in formula B, which was set at 1%, has been strategically chosen to enhance the incorporation of volatile oils. This adjustment aims to capitalize on the formula’s stability and spreading capacity, allowing for an increased concentration of active ingredients without compromising the gel’s consistency or performance. The combination of high spreading area values and excellent stability underscores the potential of formula B as an ideal candidate for further development, particularly in formulations requiring a higher load of volatile oils for enhanced therapeutic efficacy.

### 2.3. Rheological Data

**Figure 2:**
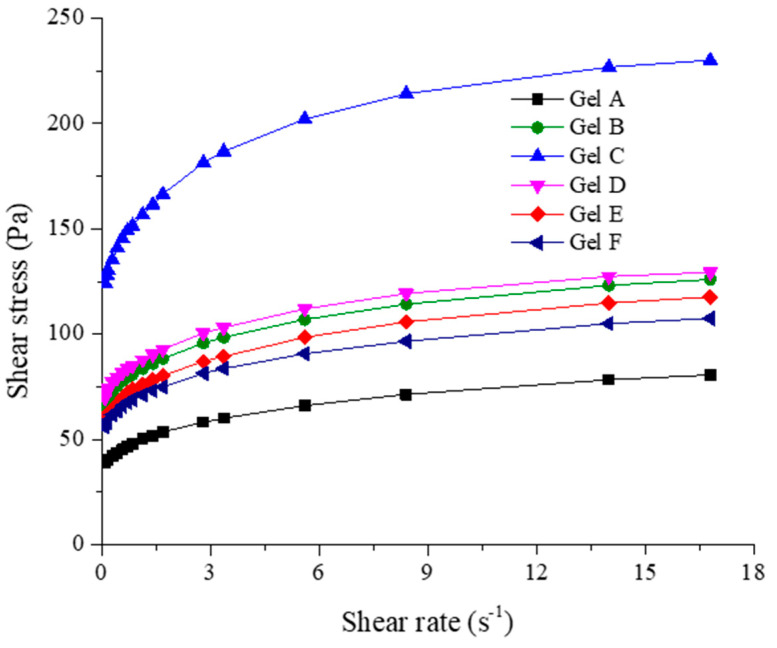
The upward rheograms for carbopol gels were analyzed at 33 °C.

For each carbopol gel, an upward rheogram was obtained by plotting the shear stress versus shear rate (Figure 2).

As can be seen from Figure 2, all formulations presented similar shapes of flow profiles with a shear stress increase for the shear rate increase, indicating a non-Newtonian behavior.

The gels’ rheological characteristics were evaluated using different models (Equations (1)–(4)), described in the Materials and Methods Section. There are two main indicators used to investigate the goodness of fit for different rheological models: the determination coefficient R^2^ and the adjusted R squared. The adjusted R^2^ takes into account the complexity of the model and provides a more accurate reflection of the model’s performance compared to the standard R^2^. This metric penalizes models for having unnecessary parameters, making it useful for comparing models with different numbers of predictors: Herschel-Bulkley model has three parameters compared to Bingham, Casson, and Ostawld-de-Waele. Which involves two parameters. The values of R^2^ and adjusted R squared of all formulations are presented in Table 5.

As can be remarked from Table 5, the R^2^ values of the Herschel-Bulkey model (Equation (4)) best fitted the experimental data, due to the highest R^2^ values ranging from 0.9947 and 0.9990 and the adjusted R squared, from 0.9939 to 0.9989. The Herschel-Bulkley model showed the highest R^2^ and adjusted R^2^ values compared to the other three models, suggesting that this rheological model is considered the best to fit the experimental data, taking into account the model complexity.

The models ranking based on accuracy alone involving the comparison of determination coefficients (R^2^) values is not enough because the variability of the model is not included. Thus, alongside R^2^ and adjusted R^2^, the model comparison and assessment were realized with the application of the Akaike Information Criterion (AIC), which estimates not only the goodness of fit, but also model variability [34]. In this study, we used the small-sample corrected AICc because the number of points was small. Therefore, the rheological model that best represents statistically the drug mechanism is the model with the lowest values of AICc [35]. According to Table 5, the lowest AICc values are recorded for Herschel-Bulkley model, which is considered to be the best model to fit the data, considering its complexity. Correlating the highest R^2^ and adjusted R^2^ values, and the smallest AICc values, we selected the best rheological model to fit the data which is represented by the Herschel-Bulkley model.

The flow parameters specific to the above model along with the viscosity recorded at 0.3 rpm, are given in Table 6.

It can be noticed that the value of the flow index (n) is smaller than 1, indicating a pseudoplastic behavior that ensures suitable gels’ manipulation and administration.

According to Table 6, the influence of composition, varying in terms of both volatile oils and carbopol concentrations, is reflected in parameters specific to the Herschel-Bulkey model and on viscosity at 0.30 rpm.

For Serie A-B-C, the concentration of gel-forming polymer is the critical ingredient affecting the rheological characteristics of the designed gels. Thus, the increase in carbopol concentration determines a significant increase of the yield stress (τ_0_), consistency index (K) and viscosity at 0.30 rpm (η_0.3_). Compared to gel A (with lower carbopol concentration), the yield stress is 1.64 times higher for gel B (with medium carbopol concentration) and 2.72 times higher for gel C (with high carbopol concentration). For a carbopol concentration varying from lower to high level, the consistency index increases 3.84 times, while the viscosity at 0.30 rpm has a 3.17 times increase. Gel B’s parameters K and η_0.3_ are about 2.20 and, respectively, 1.89 times smaller than gel C, while compared to gel A, the parameters K and η_0.3_ values are about 1.70 times higher.

For the Serie B-D-E-F, the variation of volatile oils concentration is the main factor influencing the flow parameters. All carbopol gels yield stress, consistency index, and viscosity decrease about 1.25 times when the concentration of the volatile oil varies between the minimum (2%) and maximum level (5%).

The results show that the concentration of gel-forming polymer has a stronger influence on the flow parameters, while adding essential oils modulates the gel’s rheological behavior. According to the rheological data, formulations B and E were considered the best, and the morphological and biological properties will be assessed only for these two gels. Gel B, containing 1% Carbopol, 1.5% TTO, and 1.5% RO, displayed a balanced pseudoplastic behavior with appropriate consistency and handling characteristics, making it easy to apply and spread, essential for topical treatments. The formulation showed excellent stability, maintaining its structural integrity over time. Gel E, formulated with 1% Carbopol, 2% TTO, and 2% RO, also demonstrated rheological characteristics and stability over time superior to the other formulations; in addition, it has the advantage of an increased concentration of volatile oils with a positive impact on the antimicrobial potential. Although the effect of the volatile oils on flow parameters was less pronounced than that of the gel-forming polymer, Gel E still maintained desirable rheological characteristics, ensuring ease of application and user comfort.

### 2.4. Scanning Electron Microscopy

Scanning electron microscopy (SEM) was employed to obtain detailed images of gels B and E, which were identified as the optimal formulations based on their superior rheological properties. These images provided valuable insights into the microstructure of the gels, revealing the surface morphology and the distribution of the encapsulated compounds within the hydrogel matrix. The analysis of gels B and E through SEM helped to understand how the structural characteristics of these formulations contribute to their stability, texture, and overall performance, supporting their selection as the most promising candidates for further development in topical applications.

**Figure 3:**
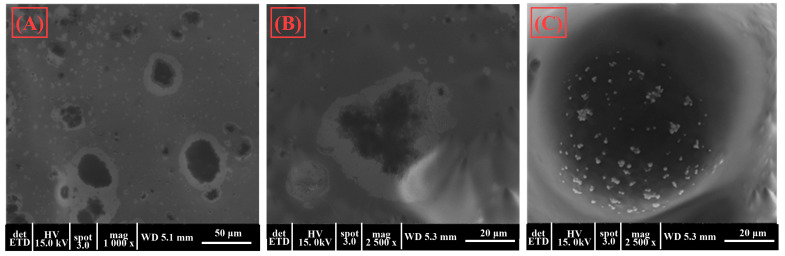
SEM images for the gel B at 1000× magnification (**A**) and 2500× magnification (**B**,**C**).

**Figure 4:**
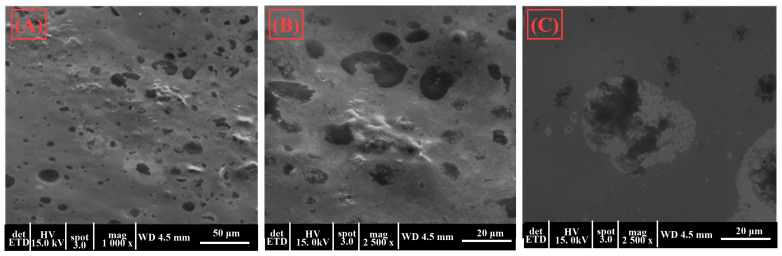
SEM images for the gel E at 1000× magnification (**A**) and 2500× magnification (**B**,**C**).

As revealed by SEM, the structural characteristics of gels B and E play an important role in determining their stability, texture, and overall performance, which are key factors in their selection as promising candidates for topical applications. The SEM images (Figure 3 and Figure 4) detailed these gels’ microarchitecture, highlighting the gel matrix’s uniformity and homogeneity. This uniform distribution of encapsulated compounds is essential for maintaining the stability of the active ingredients, as it prevents their premature release and degradation. Additionally, the fine, interconnected network observed in the gel structure contributes to its desirable texture, ensuring a smooth and consistent application on the skin. This structural integrity also enhances the gel’s ability to retain its shape and resist deformation under stress, which is critical for maintaining its rheological properties over time. The stability of the gel matrix ensures that the active ingredients are effectively preserved and delivered, while the appropriate texture promotes ease of application and user comfort. Furthermore, the well-organized microstructure facilitates a controlled and sustained release of the encapsulated compounds, which is particularly important for maintaining the antimicrobial efficacy of the gels over extended periods. This controlled release mechanism not only improves the therapeutic effectiveness of the gels but also minimizes potential side effects associated with the rapid release of active ingredients.

Overall, the structural characteristics of gels B and E, including their homogeneous distribution, stable matrix, and favorable texture, make them highly suitable for further development as topical formulations. These properties support their potential for enhanced stability, effective delivery of active compounds, and improved patient compliance, positioning them as leading candidates for innovative and effective topical therapies.

### 2.5. Antimicrobial Activity

#### 2.5.1. Qualitative Assessment of the Antimicrobial Activity

Antimicrobial activity was qualitatively evaluated by determining the growth inhibition zone diameters (GIZD) that appeared around the spot (Table 7).

Following the qualitative assessment of the antimicrobial activity, it can be seen that both the gels and the components had a variable antimicrobial effect on all the tested strains. Larger growth inhibition zone diameters of the same strains were obtained for the gel components compared to the inhibition diameters obtained for the gels. These results were expected considering that one of the reasons for incorporating the bioactive components into the polymeric matrix was their gradual release, thus ensuring a prolonged effect.

For some strains (*S. aureus* 14, *S. aureus* 20, *S. aureus* 25, *S. aureus* 30, *S. aureus* 34, *S. aureus* 38, *S. aureus* 540) the growth inhibition was observed through the lower density of bacterial colonies compared to the areas where the test product was not applied. However, the inhibition diameter of bacterial growth could not be measured. Therefore, quantitative testing was performed to quantify the growth inhibition activity against these strains.

#### 2.5.2. Quantitative Assessment of the Antimicrobial Activity

**Figure 5:**
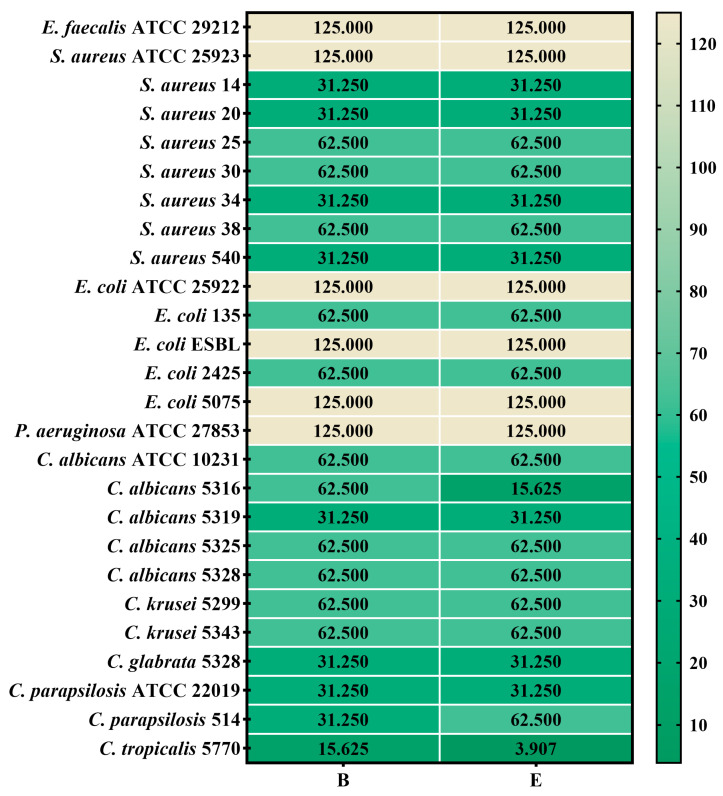
Heatmap of MIC values (mg/mL) for gels B and E. The scale bar displays the variations in the sensitivity of the strains from the highest (green forest) to the lowest (light vanilla). Legend: GB = gel base; B = formula B; E = formula E; CO = clove essential oil; RO = Rosemary essential oil; TTO = tea tree essential oil; UA = Usnic acid; Complex = Cyclodextrin complex with CO. The influence of the formulas B and E on each microbial strain was statistically analyzed using one-way ANOVA and Tukey’s multiple comparisons test. The data results were statistically significant (*p* < 0.05).

**Figure 6:**
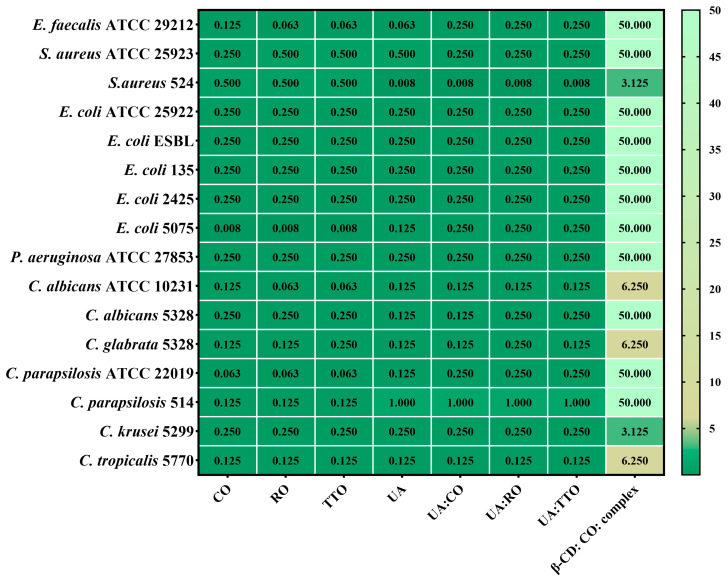
Heatmap of bioactive components’ MIC values (mg/mL) from gel formulations. Legend: Clove essential oil = CO; Rosemary essential oil = RO; tea tree essential oil = TTO; Usnic acid = UA; complex = Cyclodextrin complex with CO; β-CD = β-Cyclodextrin. The scale bar displays the variations in the sensitivity of the strains from the highest (green forest) to the lowest (light vanilla). The influence of the bioactive compounds on each microbial strain was statistically analyzed using one-way ANOVA and Tukey’s multiple comparisons test. The data results were statistically significant (*p* < 0.05).

The quantitative assessment of antimicrobial activity involves establishing the minimum inhibitory concentration (MIC), the lowest concentration of the antimicrobial agent that prevents the visible growth of a microorganism. The MIC results are presented in Figure 5 for gels B and E, and Figure 6 displays the MIC results for their components.

The data obtained demonstrated that both gel formulations exhibited antimicrobial activity against Gram-positive bacteria, Gram-negative bacteria, and yeasts from *Candida* spp., cultured from standardized ATCC strains or isolated from biological samples (Table 7). Although the antimicrobial activities of the two formulations (B and E) were generally comparable, specific differences were evident. The MICs ranged from 31.250 to 125.000 mg/mL for bacterial strains and from 3.907 to 62.500 mg/mL for yeasts. Both formulations showed similar activity, but formulation E exhibited superior efficacy against *C. tropicalis* 5770, with a MIC as low as 3.907 mg/mL, compared to formulation B, which had a MIC of 15.625 mg/mL. This enhanced activity in formulation E is likely due to its higher content of TTO and RO, known for their potent antimicrobial properties (Figure 5 and Figure 6). Despite the differences, the similar antimicrobial activity observed against the other microbial strains may be attributed to the identical concentration of Carbopol 940 in both formulations, as well as the similar inclusion of the cyclodextrin complex. These factors could result in comparable release kinetics of the bioactive compounds, thus leading to similar levels of antimicrobial effectiveness [36]. These results encouraged us to continue testing the gels using a larger number of bacterial strains isolated from the clinic.

#### 2.5.3. Semiquantitative Assessment of the Microbial Adherence to the Inert Substratum

The semiquantitative assessment of microbial adherence to the inert substratum involved evaluating the microorganisms’ ability to attach and form biofilms on non-biological surfaces used to mimic medical devices or other non-living materials [37]. The minimum biofilm eradication concentration (MBEC) is critical in evaluating antimicrobial agents’ effectiveness, mainly when dealing with biofilm-associated infections that are challenging to treat. In our study on hydrogels containing cyclodextrin complexes with volatile oils, MBEC plays a vital role in determining the lowest concentration of the formulation needed to eradicate biofilms formed by pathogens completely. Biofilms provide a protective barrier for microorganisms, making them more resistant to treatment than planktonic (free-floating) bacteria. By assessing MBEC, the study offers new insights into the real-world efficacy of these hydrogels against resilient biofilm-forming pathogens, ensuring that the formulations are effective against free-floating microbes (sometimes resistant strains) and capable of penetrating and eradicating biofilms.

**Figure 7:**
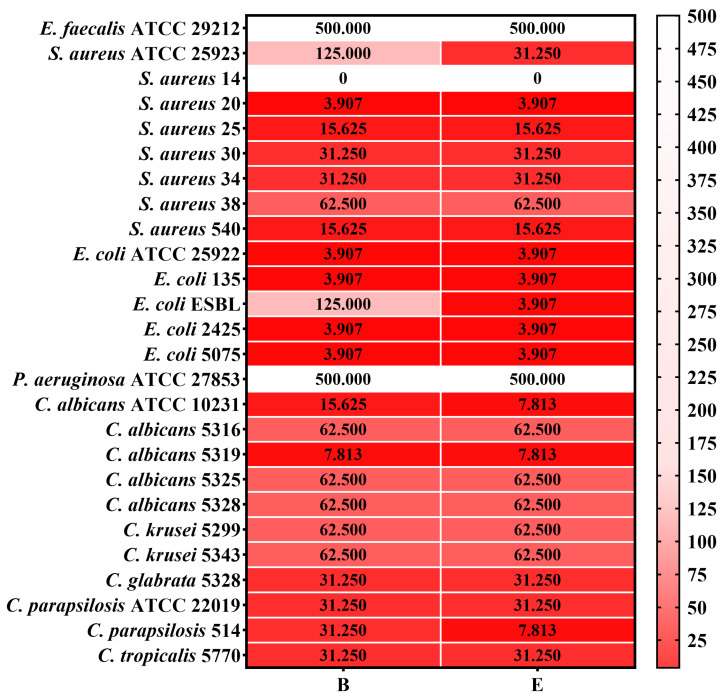
Heatmap of MBEC values (mg/mL) for gel formulations B and E. The scale bar displays the variations in the sensitivity of the strains from the highest (intense red) to the lowest (light red). Legend: B = gel formulation B; E = gel formulation E. The scale bar displays the variations in the sensitivity of the strains from the highest (intense red) to the lowest (white). The influence of the formulas B and E on the adherence of each microbial strain tested was statistically analyzed using one-way ANOVA and Tukey’s multiple comparisons test. The data results were statistically significant (*p* < 0.05).

**Figure 8:**
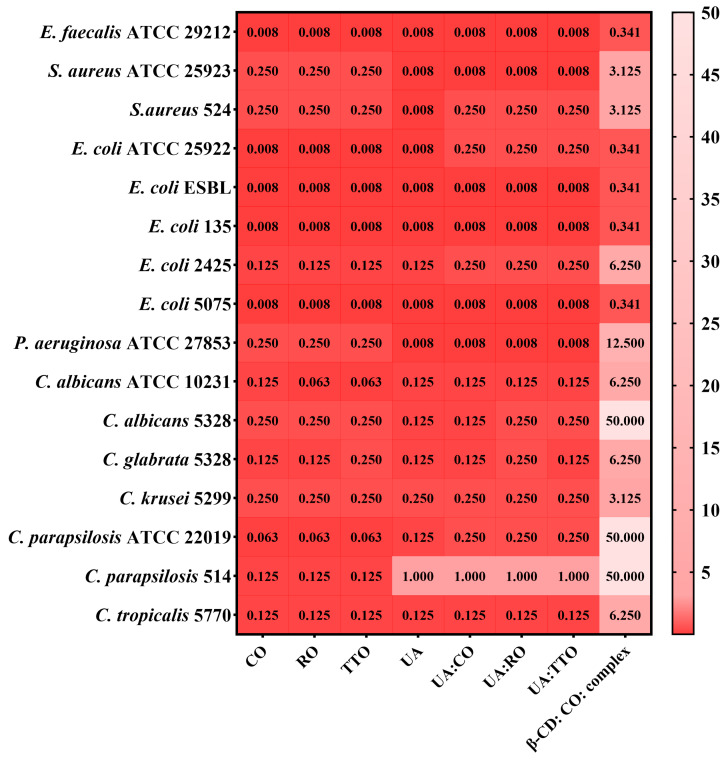
Heatmap of MBEC values (mg/mL) for bioactive components from gel formulations. Legend: Clove essential oil = CO; Rosemary essential oil = RO; Tea tree essential oil = TTO; Usnic acid = UA; Complex = Cyclodextrin complex with CO; β-CD = β-Cyclodextrin. The scale bar displays the variations in the sensitivity of the strains from the highest (intense red) to the lowest (white). The influence of the bioactive compounds on each microbial strain’ adherence was statistically analyzed using one-way ANOVA and Tukey’s multiple comparisons test. The data results were statistically significant (*p* < 0.05).

The MBEC results are presented in Figure 7 for gels B and E, and Figure 8 displays the MBEC results for their components.

However, by evaluating microbial adherence to an inert substrate, neither formulation significantly affected the *S. aureus* 14 clinical strain. Additionally, higher concentrations of both products were required to inhibit microbial adherence in the cases of *E. faecalis* ATCC 29212 and *P. aeruginosa* ATCC 27853, indicating that while the gels are effective in inhibiting microbial growth, their impact on microbial adherence may be limited and strain-specific (Figure 7 and Figure 8).

The literature data confirm the inhibitory effect of tea tree, rosemary, and clove essential oils against the growth of bacterial and yeast strains. These oils are suitable especially for wound infections produced by *S. aureus*, *S. epidermidis*, *S. pyogenes*, *E. faecalis*, *E. coli*, *C. albicans* [38,39,40,41].

In a study from 2012, Sienkiewicz et al. [42] demonstrated the antimicrobial activity of RO on *E. coli* (ESBL) clinical strains isolated from the abdominal cavity. Additional studies have shown the antibacterial properties of RO against *E. coli*, *Bacillus cereus*, *S. aureus*, *Clostridium perfringens*, *Aeromonas hydrophila*, and *Salmonella choleraesuis* [43]. On the other hand, Fabio et al. [44] reported that RO and CO exhibit antibacterial effects against various microorganisms responsible for respiratory infections isolated from some clinical samples. These include both antibiotic-sensitive and antibiotic-resistant strains such (*S. agalactiae*, *S. pyogenes*, *S. pneumoniae*, *S. aureus*, *Klebsiella pneumoniae* and *Stenotrophomonas maltophilia*).

Studies have also shown that TTO has the ability to inhibit the growth of some bacterial strains susceptible to antibiotic resistance (*E. coli* strain AG100 and *S. aureus* NCTC 8325) by increasing membrane permeability and inhibiting respiration in microbial cells. Furthermore, some studies mention the ability of Carbopol^®^ Ultrez hydrogels with TTO nanoemulsions and nanocapsules improve wound healing and possess an antiedematogenic effect [45].

The antimicrobial activity of TTO and CO was also highlighted by Altaf et al. [46], who incorporated them into PVA/starch hydrogels.

In addition to the fact that *in vitro* antibacterial analysis of TTO-loaded hydrogels (using the disc diffusion method) demonstrated good antibacterial activity against *E. coli* and multi-resistant *S. aureus* (MRSA), they also have the capacity to accelerate the process of wound healing. However, it was not more effective than CO-loaded hydrogels [46].

The antimicrobial efficacy of the volatile oils against bacterial strains and yeasts may be attributed to the synergistic effects of their various constituents within the volatile fraction, rather than the influence of any single component. For instance, a study on the antimicrobial activity of terpinen-4-ol (the primary constituent of TTO) showed that *S. aureus* strains isolated from biological samples (nasal and oropharyngeal secretions) are sensitive. Furthermore, terpinen-4-ol demonstrated a significant ability to prevent the biofilm formation of these strains by targeting the penicillin-binding protein 2a [47]. Likewise, Zhang et al. [48] demonstrated that terpinen-4-ol affected the cell membrane and wall of *Streptococcus agalactiae* by increasing the cell membrane permeability and by releasing lactate dehydrogenase. Another study indicated that components of the TTO (terpinen-4-ol, α-terpineol, terpinolene, linalool, cineole, α-terpinene, γ-terpinene) had variable actions against *B. subtilis*, *C. albicans*, *Bacteroides fragilis*, *Clostridium perfringens*, *E. faecalis*, *E. coli*, *Lactobacillus acidophilus*, *Moraxella catarrhalis*, *Mycobacterium smegmatis*, *Serratia marcescens* and *S. aureus*) [49].

Eucalyptol, the significant component of RO, is also known as an antimicrobial agent and has attracted considerable interest for its potential to reduce antibiotic resistance. Studies have shown that it is effective against Gram-positive strains (*S. aureus* and *B. subtilis*), Gram-negative strains (*E. coli* and *P. aeruginosa*) and yeasts (*C. albicans* and *C. glabrata*) [50,51,52,53,54,55,56]. The primary mechanism of action of eucalyptol is based on the disruption of membrane integrity, alteration of surface charge, and generation of reactive oxygen species (ROS)/oxidative stress [57]. Also, eucalyptol is capable of downregulating genes related to carbohydrate metabolism and membrane proteins at the mRNA level. Moreover, at the fungal level, eucalyptol downregulated ergosterol biosynthetic genes, inhibited biofilm formation, generated ROS, caused cell cycle arrest at the G1/S phase, and altered the mitochondrial membrane [58].

The main component of CO, eugenol, has been reported in the literature as both a bactericidal and bacteriostatic agent, also possessing antifungal properties. The antimicrobial mechanism of action of eugenol has been attributed to the free OH group in its molecular structure. Moreover, eugenol damages the cytoplasmatic membrane and generates the leakage of intracellular components [59]. It has shown inhibitory effects on the growth of several strains, including *E. faecalis*, *S. pyogenes*, *S. aureus*, *S. pneumoniae*, *Salmonella choleraesuis*, *Yersinia enterocolitica*, *Helicobacter pylori*, *P. aeruginosa*, *E. coli* [60,61,62,63]. The antifungal activity of eugenol has also been reported against *C. albicans*, *C. tropicalis*, and *C. krusei* [63,64,65].

Moreover, it is well known that usnic acid exhibits antimicrobial activity. In a 2007 study, Elo et al. [66] examined the in vitro antimicrobial activity of usnic acid and its sodium salt against clinical isolates of vancomycin-resistant enterococci (VRE) and methicillin-resistant *S. aureus* (MRSA). The results showed that usnic acid, particularly its sodium salt, exhibited strong antimicrobial activity against all tested clinical isolates. In addition, *Vibrio harveyi* and *B. subtilis* have been found to be sensitive to usnic acid at various concentrations [67]. The growth inhibition of *C. orthopsilosis* and *C. parapsilosis* strains, and *C. albicans* biofilms demonstrates that usnic acid can be a good candidate for formulating pharmaceutical products for treating candidiasis [68,69].

It can also be observed that the bioactive components have both MIC and MBEC values lower when they were tested as such compared to the gels in which they were incorporated. This aspect highlights the ability of the carbopol matrix to gradually release the volatiles compounds from the TTO and RO as the first antimicrobial barrier, as well as the gradual release of the CO from the complex and then its delivery into the biological environment. This data focus attention to the potential of the gels to provide antimicrobial protection for a period greater than 24 h.

### 2.6. Cytotoxic Activity

**Figure 9:**
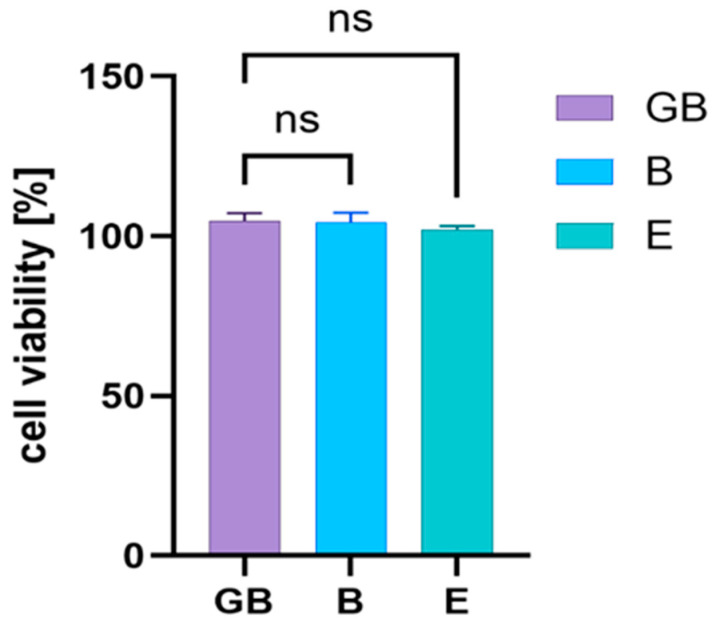
The gels’ effect on the viability of the antitumoral cells. Legend: GB = gel base; B = formula B; E = formula E; ns = not significant. The gels’ influence was statistically analyzed using two-way ANOVA followed by Dunnett’s multiple comparisons tests, which did not reveal statistically significant correlations.

The impact of pharmaceutical formulations B, E, and the gel base (GB) on cell viability and proliferation of the Human Colorectal Adenocarcinoma Cell Line (ATCC HTB-38) was assessed using the MTT assay (Figure 9).

The essential oil components added to GB did not modify HTB-38 tumoral cell proliferation in the 1% concentration gels tested. On the other part, values close to 100% indicate that most cells survived the treatment with the respective gels, and their cytotoxicity is relatively low at the 1% concentration, where no statistically significant changes are observed.

Other types of complex BCD-based also proved to be non-toxic [70]. Toxicity studies on a curcumin-loaded hydrogel with formulation materials based on 2-hydroxypropyl-β-CD, sodium alginate, and chitosan designed for wound dressing materials with antimicrobial activity, tested on NCTC clone 929 cells and normal human dermal fibroblasts, showed the hydrogel was non-toxic.

A doxorubicin-loaded hydrogel containing β-CD and agarose did not exhibit toxicity toward HEK-293 (Human Embryonic Kidney) and HeLa cells [71].

Otherwise, the majority of the inclusion complexes of LCEO (*Litsea cubeba* essential oil) into β-CD (β-cyclodextrin) exhibited antitumor activity against the tumor (HT-29 and HeLa) and non-tumor (Vero) cell lines by MTT assay [72].

## 3. Conclusions

The composition of the designed gels influences the flow parameters, which is more evident for the carbopol concentration variation. All hydrogels exhibited non-Newtonian pseudoplastic behavior with yield stress. The rheological evaluation is paramount for a complex physico-chemical evaluation of semisolid dosage forms, providing valuable information concerning their fabrication technology, quality and stability control during preservation, and therapeutical activity. This study developed and evaluated hydrogels containing a cyclodextrin complex with CO alongside TTO and RO, focusing on their pharmaco-technical and rheological properties. The results demonstrate that the concentration of Carbopol 940 is the primary factor influencing the gels’ rheological behavior, with a higher concentration leading to increased yield stress, consistency index, and viscosity. Formula B, containing 1% Carbopol, 1.5% TTO, and 1.5% RO, exhibited optimal stability and rheological properties, making it suitable for practical application. In contrast, Formula E, with the same Carbopol concentration but higher amounts of TTO (2%) and RO (2%), displayed superior antimicrobial efficacy, particularly against *Candida tropicalis*. This enhanced activity is attributed to the increased levels of free volatile oils. The study underscores the importance of balancing the polymer and volatile oil concentrations to optimize the hydrogels’ physical properties and antimicrobial effectiveness, highlighting their potential for medical and cosmetic applications. Based on this study, the proof of concept was laid down and further studies will be done for a complete characterization considering the desired pharmaceutical and therapeutic applications.

The current focus on topical applications could be expanded to explore other therapeutic areas, such as mucosal drug delivery, ophthalmic formulations, or vaginal/rectal delivery. Hydrogels are ideal candidates for these applications due to their ability to adhere to mucosal surfaces and provide localized drug delivery with minimal systemic absorption. Research into biodegradable hydrogels for tissue engineering or regenerative medicine could also be an important future direction, exploring how these formulations can serve as scaffolds for cell growth or as delivery vehicles for growth factors and stem cells in tissue repair.

To fully unlock the potential of these hydrogels in pharmaceutical and therapeutic applications, future research should focus on more advanced characterization methods that assess drug release behavior, biocompatibility, therapeutic efficacy, and mechanical properties under conditions mimicking their intended use. Such studies will significantly enhance the clinical relevance of the findings and open up broader opportunities for the application of these hydrogels in medicine and healthcare.

## 4. Materials and Methods

### 4.1. GC-MS Analysis

The chemical composition of essential oils from *Melaleuca alternifolia* (tea tree) and *Rosmarinus officinalis* (rosemary) was analyzed using a Focus gas paired with a Polaris Q ion trap mass spectrometer and a Triplus autosampler (Thermo Fisher Scientific, Waltham, MA, USA). The system utilized a DB-5MS capillary column (25 m length, 0.25 mm diameter, and 0.25 μm film thickness). The temperature program for the oven started at 60 °C for 3 min, increased at a rate of 10 °C per minute up to 200 °C (held for 2 min), and then further increased at 12 °C per minute to a final temperature of 240 °C. Helium was used as the carrier gas with a flow rate of 1 mL/min. The ion source and interface temperatures were set to 200 °C and 250 °C, respectively, with the mass spectrometer operating in electron impact mode at 70 eV. Detection was carried out within an *m*/*z* range of 35–300, and the mass spectrometer was run in full-scan mode. Chromatogram analysis was performed using X calibur software 4.3 (Thermo Fisher Scientific, Waltham, MA, USA)., supported by the NIST 11 database for compound identification. An alkane standard solution for GC (C8-C20 in hexane, Sigma-Aldrich, Darmstadt, Germany) was used to determine Kovats indices (KIs), the percentage composition of the identified compounds being calculated from the total ion chromatogram based on GC peak areas.

The oil samples were diluted with hexane to volume ratio 1:10.

### 4.2. Formulation of Hydrogels

The objective of this study was the development and pharmaco-technical characterization, especially rheological, of volatile oil-based hydrogels. For the formulation of gels with antimicrobial action, two levels of variation of the composition were chosen: the concentration of hydrophilic polymer (Carbopol 940, Sigma-Aldrich, Darmstadt, Germany) in the gel base (Table 8) and the concentration of volatile oils in the final formula (Table 9).

For the preparation of semisolid hydrogel pharmaceutical forms, the polyacrylic type network (Carbopol 940, corresponding to a final concentration of 0.8, 1, respectively, 1.2%) was hydrated using 75% of the amount of purified water provided in the formula for at least 24 h in the presence of glycerin (purity over 99%; Glycerin from Merck, Darmstadt, Germany) used as a dispersing agent. Triethanolamine (purity over 99%; Triethanolamine from Carl Roth GmbH + Co. KG., Karlsruhe, Germany) was used for neutralization at the end of this interval (Table 8). The remaining amount of water was dispersed with cyclodextrin complex with CO (synthesized in the laboratory) and added to the semisolid matrix generated by hydration of the hydrophilic polymer under intense stirring (2000 rpm for 10 min, using a turbine stirrer in a Heidolph RZR 2020, Heidolph Instruments GmbH and Co. KG, Schwabach, Germany). Finally, the usnic acid (purity over 98%; from Merck, Darmstadt, Germany) was mixed with the volatile oils and added to the semisolid matrix under stirring until complete homogenization. The volatile oils used to prepare gels are organic products from Mayam, with a quality certificate (certified by Ecocert Greenlife according to the Ecocert/Cosmos standard). The TTO and RO have antimicrobial action and ensure good preservation of the gels.

In the second case, gels with Carbopol 940 1% were made in which the concentration of volatile oils represented 2%, 4%, and 5% in the total mass of the preparations (Table 9). Purified water (conductivity below 0.05 µS/cm) was obtained in a system Evoqua Ultra Clear TP ED ultrapure water system, Evoqua, Berlin, Germany. The remaining reagents used were purity analytical (Merck, Darmstadt, Germany).

Carbopol 940 is a polymer of carboxyvinyl that appears as a white, light powder, partially soluble in water, and soluble in solutions of alkaline hydroxides and amines. Glycerol is a syrupy liquid, clear, colorless, odorless and sweet in taste. It is hygroscopic, miscible with alcohol and water, and insoluble in fatty and volatile oils.

Due to the strong hydrophilic character of carbopol, it tends to form agglomerations that are difficult to disperse later when introduced into water, so the carbopol is slowly introduced into water and under continuous agitation. Stirring is continued until an opalescent dispersion is obtained without agglomeration. The soaking stand can be extended up to 24 h, during which time any air incorporated during stirring is removed. When triethanolamine is added, the acid groups of carbopol are neutralized, simultaneously with its dissolution in water and gelation of the dispersion.

The spreading capacity of the gel formulations was measured 48 h after preparation by measuring the spreading diameter of 1 g of the gel between two 20 × 20 cm glass plates after 1 min. The mass of the upper plate was standardized at 125 g. Weighs of 50 g, 100 g, 200 g and 250 g were subsequently placed over the sample at 1 min. intervals. The spreading areas reached by the sample were measured in millimeters in the vertical and horizontal axes.

The results were expressed in terms of the spreading area as a function of the applied mass according to the following equation:

S_i_ = d_i_^2^ (π/4), in which S_i_ is the spreading area (mm^2^) resulting from the applied mass i (g) and d_i_^2^ is the mean diameter (mm) reached by the sample [73].

The gels were also submitted to some control tests to determine their characteristics and stability:

- The determination of the appearance was made by examination with the magnifying glass (4.5×) of a sample stretched by a thin layer on a microscopic blade;
- The determination of the main characteristics was made according to Romanian Pharmacopoeia Xth edition [74];
- pH was determined after the gel samples were suitably processed, respectively after the dispersion of samples in water (1:5), followed by the measuring of the pH of an aqueous phase, as follows: 25 mL of distilled water were added to 5 g sample and the mixture was stirred into an Erlenmeyer glass with cork heating on the water bath at 60 °C for 10 min. After cooling on the watery phase, the pH was determined with Radelkis pH meter (Radelkis Ktsz.; Budapest; Hungary);
- The determination of the viscosity was performed with a rotational Brookfield LFV viscometer (AMETEK Brookfield, Middleboro, MA, USA);
- The stability was performed by maintaining the samples at 2 °C and 40 °C: 5 g of sample were introduced into a weighing ampoule with a lid, and then, the ampoule was maintained for 8 h at mentioned temperatures. Then, the appearance of the sample was examined.

The gels’ compositions were presented in Table 8 and Table 9, and gel base (GB) is considered the formulation without bioactive compounds (cyclodextrin complex with volatile oil, usnic acid, or volatile oils).

### 4.3. Rheological Analysis and Data Modeling

The rheological behavior of the designed carbopol gels was evaluated through stationary shear analysis at 33 °C ± 0.1 °C (to simulate the skin temperature), using a rotational viscometer Multi-Visc Rheometer (Fungilab, Madrid, Spain). The measuring system was equipped with a ThermoHaake P5 Ultrathermostat to maintain constant the sample temperature during the experiment. Our previous works detailed the operating conditions [75,76]. Briefly, after mechanical and thermal equilibration, the gels were sheared at a shear rate specific to the TR 10 standard spindle, from 0.08 to 16.8 s^−1^, corresponding to a rotational speed between 0.30 and 60 rpm. The shear stress as a function of shear rate upward rheograms was obtained. The rheological data were further analyzed by applying different shear stress (τ) versus shear rate ($\dot{\gamma }$) models as follows: Bingham (Equation (1)), Casson (Equation (2)), Ostwald-de Waele (Equation (3)) and Herschel-Bulkley (Equation (4)):

(1) $$\tau =\tau_{0}+\eta \cdot \dot{\gamma }$$

(2) $$\tau^{0.5}={\tau_{0}}^{0.5}+\eta^{0.5}\cdot {\dot{\gamma }}^{0.5}$$

(3) $$\tau =K\cdot {\dot{\gamma }}^{n}$$

(4) $$\tau =\tau_{0}+K\cdot {\dot{\gamma }}^{n}$$

where η is plastic viscosity (Pa·s), τ_0_—yield stress (Pa), K—consistency index (Pa·s^n^), and n—flow index (dimensionless) [76,77,78]. The flow parameters were determined using Table Curve 2D software 5.1, Systat Software (Palo Alto, CA, USA).

### 4.4. SEM Analysis

Surface morphology of the samples was obtained using an FEI Nova NanoSEM 630 scanning electron microscope (Nanotech, Tokyo, Japan) with a TLD (Through-Lens-Detector), at an acceleration voltage of 5 kV and a working distance of 4.7 mm.

### 4.5. Antimicrobial Activity

The antimicrobial assessments were performed with standard strains (*Enterococcus faecalis* ATCC 29212, *Staphylococcus aureus* ATCC 25923, *Escherichia coli* ATCC 25922, *Pseudomonas aeruginosa* ATCC 27853, *Candida albicans* ATCC 10231, and *Candida parapsilosis* ATCC 22019. In addition, clinically isolated strains from skin and mucous infections (*Staphylococcus aureus* 14, *Staphylococcus aureus* 20, *Staphylococcus aureus* 25, *Staphylococcus aureus* 30, *Staphylococcus aureus* 34, *Staphylococcus aureus* 38, *Staphylococcus aureus* 540, *Escherichia coli* 135, *Escherichia coli* 2425, *Escherichia coli* 5075, *Escherichia coli* ESBL, *Candida albicans* 5316, *Candida albicans* 5319, *Candida albicans* 5325, *Candida albicans* 5328, *Candida krusei* 5299, *Candida krusei* 5343, *Candida glabrata* 5328, *Candida parapsilosis* 514 and *Candida tropicalis* 5770) were also used. All strains tested are provided from the Microorganisms Collection of the Department of Microbiology, Faculty of Biology and Research Institute of the University of Bucharest.

#### 4.5.1. Qualitative Assay of the Antimicrobial Activity

The inhibitory effects of the gels were assessed using a spot diffusion method and according to the Clinical Laboratory Standards Institute [79,80]. Bacterial and yeast suspensions (1.5 × 10^8^ CFU/mL and 3 × 10^8^ CFU/mL, respectively) were obtained from 24 h cultures on Nutrient Broth No. 2 (NB) and Sabouraud Glucose Agar with chloramphenicol (Sab) medium. Petri plates with the specific media were seeded with inoculums, and each 20 µL of each sample was spotted. The negative control was considered the sterile medium, and the positive control was the NB/Sab medium inoculated with microbial suspensions. After diffusion, the dishes were incubated at 37 °C for 24 h for bacteria and 48 h for yeast strains.

#### 4.5.2. Quantitative Assay of the Antimicrobial Activity

The minimum inhibitory concentration (MIC) assessment was performed using an adapted binary serial microdilution standard assay in liquid media utilizing 96-well microtiter plates. From each sample, serial two-fold micro-dilutions were realized in 150 µL of corresponding broth medium seeded with the standard inoculum. The microtiter plates were incubated at 37 °C for 24 h. Visual and spectrophotometric analyses determined the MIC values by measuring the absorbance at 620 nm via the BIOTEK SYNERGY-HTX ELISA multi-mode reader (Winooski, VT, USA) [79].

#### 4.5.3. Semiquantitative Assessment of the Microbial Adherence to the Inert Substratum

The biofilm development on the inert substratum was assessed utilizing the same serial two-fold microdilution method. Following 24 h of incubation, the medium from microtiter plates (containing binary dilutions of the samples) was removed, the wells were washed three times with sterile physiological buffer saline, and the bacterial cells adhered to the walls were fixed with methanol (5 min) and tinted with 1% crystal violet (15 min). The stained biofilm was resuspended with 33% acetic acid, and the absorbance was measured at 490 nm [79].

### 4.6. Cytotoxic Activity

The cytotoxicity of the gels was assessed by determining the viability of a Human Colorectal Adenocarcinoma Cell Line (ATCC HTB-38) using the MTT assay (Sigma, Santa Clara, CA, USA). HTB-38 cells were cultured in RPMI 1640 medium (Lonza, Basel, Switzerland) supplemented with 10% fetal bovine serum (FBS) and Pen-Strep (Biochrom, Berlin, Germany) in 96-well polystyrene plates. The cells were incubated at 37 °C in a 5% CO_2_ environment until they reached 70% confluence (passage 48). The medium was then removed, and the cells were exposed to 100 µL of RPMI 1640 medium containing 10% FBS, Pen-Strep, and the gel samples for 24 h under the same conditions. After incubation, the medium and biomaterials were removed, and the cells were washed twice with warm PBS before being treated with 100 µg/mL MTT solution for 4 h at 37 °C in a 5% CO_2_ atmosphere. Following incubation, the dye was dissolved in dimethyl sulfoxide (DMSO), and absorbance was measured at 540 nm using a Synergy HTX spectrophotometer (Agilent Technology Biotek, Santa Clara, CA, USA).

### 4.7. β-cyclodextrin-Eugenia caryophyllata Volatile Oil Complex Preparation

The encapsulation of *Eugenia caryophyllata* (clove) volatile oil was achieved through a methodical process to ensure optimal inclusion within the β-cyclodextrin matrix. Initially, β-cyclodextrin was dissolved in a water/ethanol solution (3:1/*v*:*v*), providing an appropriate medium for the complexation process. The volatile oil was then gradually introduced to the solution at room temperature, maintaining constant stirring to promote uniform dispersion and interaction between the cyclodextrin and the oil. The mixture adhered to a precise mass ratio of 4:1/*w*:*w* (β-cyclodextrin/volatile oil), a ratio selected to maximize encapsulation efficiency while preserving the stability of the complex. Following the formation of a homogeneous suspension, the mixture underwent lyophilization—a freeze-drying process that gently removed the solvent without compromising the integrity of the encapsulated oil. This method yielded a dry, stable complex in powder form, suitable for further application in various pharmaceutical formulations [81,82,83].

### 4.8. β-cyclodextrin-Eugenia caryophyllata Volatile Oil Complex Characteristics

The β-cyclodextrin-*Eugenia caryophyllata* (clove) volatile oil complex was successfully obtained as a compact white powder with a distinctive clove-like aroma, indicating the presence of the encapsulated volatile oil. The encapsulation efficiency was notably high (83.35%), which corresponding to approximately 170 mg of volatile oil per gram of the complex, reflecting the effectiveness of the β-cyclodextrin in trapping the volatile components. This encapsulation is essential for protecting the oil’s active compounds from degradation, enhancing their stability, and potentially improving their bioavailability in subsequent applications. The detailed analysis and comprehensive characterization of this complex, including its physicochemical properties, thermal stability, and potential interactions within the matrix, will be thoroughly explored in a forthcoming publication, which constitutes the second part of this study.

### 4.9. Statistical Analysis

The data results were statistically analyzed with GraphPad Prism 10.3 from GraphPad Software, San Diego, CA, USA. All experiments were performed in three independent determinations. The results are expressed as ±SD (standard deviation) and analyzed using a one-way analysis of variance followed by a multiple comparisons assay agreeing to the experiment method. The differences between groups/samples were considered statistically significant when the *p*-value was <0.05.

## Figures and Tables

**Table 1:** The chemical composition of *Melaleuca alternifolia* essential oil.

Compounds	KI *	t_R_ (min) **	Relative Area (%)
α-Pinene	930	6.01	1.02
Sabinene (β-Thujene)	969	6.72	0.72
β-Pinene	982	6.95	0.45
α-Phellandrene	997	7.23	0.27
**α-Terpinene**	1009	7.44	10.85
**p-Cymene**	1017	7.58	2.63
**Eucalyptol**	1026	7.73	3.76
**γ-Terpinene**	1051	8.17	18.77
**α-Terpinolene**	1078	8.64	3.43
trans-Sabinene hydrate	1090	8.84	0.15
cis-2-p-Menthen-1-ol	1113	9.21	0.43
Borneol	1162	9.99	0.01
**Terpinen-4-ol**	1175	10.20	f36.32
**α-Terpineol**	1183	10.32	2.63
cis-Piperitol	1197	10.54	0.17
Geraniol	1238	11.15	0.01
δ-EIemene	1324	12.39	0.06
α-Cubebene	1336	12.55	0.09
Eugenol	1344	12.66	0.08
α-Copaene	1364	12.94	0.28
Methyleugenol	1384	13.23	0.09
α-Gurjunene	1398	13.42	0.75
β-Caryophyllene	1409	13.56	0.41
γ-Elemene	1430	13.82	1.95
Alloaromadendrene	1454	14.11	0.75
γ-Muurolene	1463	14.22	0.91
β-Guaiene	1480	14.43	0.53
**α-Muurolene**	1490	14.56	3.88
**γ-Cadinene**	1512	14.82	3.48
δ-Cadinene	1523	14.95	0.38
Spathulenol	1574	15.56	0.27
Globulol	1582	15.65	0.71
Viridiflorol	1590	15.75	0.63
Rosifoliol	1597	15.84	0.15
Ledol	1601	15.88	0.10
Cubenol	1614	16.12	0.55
epi-Cubenol	1621	16.25	0.15
τ-Cadinol	1623	16.29	0.45
Total			98.27

* KI-Kovats Index, ** t_R_—retention time.

**Table 2:** The chemical composition of *Rosmarinus officinalis* essential oil.

Compounds	KI *	t_R_ (min) **	Relative Area (%)
**α-Pinene**	932	6.03	11.83
**Camphene**	946	6.29	3.93
**Sabinene (β-Thujene)**	973	6.79	4.10
β-Pinene	982	6.96	0.71
α-Phellandrene	998	7.26	0.11
p-Cymene	1011	7.48	0.03
**Eucalyptol**	1030	7.80	49.70
γ-Terpinene	1049	8.14	0.52
cis-Sabinene hydrate	1060	8.33	0.04
α-Terpinolene	1078	8.64	0.35
Linalool	1087	8.79	0.63
endo-Fenchol	1107	9.12	0.03
α-Campholenal	1116	9.26	0.04
**Camphor**	1143	9.69	14.12
**Borneol**	1161	9.98	3.36
Terpinen-4-ol	1169	10.10	0.56
**α-Terpineol**	1183	10.32	2.00
Myrtenol	1189	10.41	0.04
Verbenone	1204	10.65	0.01
cis-Carveol	1208	10.71	0.01
Thymol	1271	11.62	0.02
Bornyl acetate	1275	11.68	0.75
Carvacrol	1287	11.86	0.02
Eugenol	1344	12.66	0.02
α-Ylangene	1359	12.88	0.03
α-Copaene	1364	12.94	0.02
Methyleugenol	1389	13.29	0.03
**β-Caryophyllene**	1412	13.60	5.80
β-Ylangene	1418	13.67	0.05
α-Guaiene	1431	13.83	0.03
Aromadendrene	1439	13.93	0.03
α-Humulene	1446	14.01	0.42
γ-Muurolene	1465	14.25	0.04
α-Muurolene	1491	14.57	0.15
γ-Cadinene	1511	14.81	0.03
α-Calacorene	1535	15.10	0.01
Caryophyllene oxide	1582	15.65	0.24
Methyl jasmonate	1621	16.26	0.04
Total			99.84

* KI-Kovats Index, ** t_R_—retention time.

**Table 3:** Gel characteristics.

Characteristic	Formula A	Formula B	Formula C
**Initial macroscopic characteristics**	appearance: homogenous; color: yellowish; smell: specific	appearance: homogenous; color: yellowish; smell: specific	appearance: homogenous; color: yellowish; smell: specific
**Macroscopic characteristics after 60 days**	appearance: homogenous; color: yellowish smell: specific	appearance: homogenous; color: yellowish; smell: specific	appearance: homogenous; color: yellowish; smell: specific
**Initial pH**	6.50	6.00	6.70
**pH after 60 days**	6.40	6.60	6.60

**Table 4:** Gel characteristics.

Characteristic	Formula D	Formula E	Formula F
**Initial macroscopic characteristics**	appearance: homogenous; color: yellowish; smell: specific	appearance: homogenous; color: yellowish; smell: specific	appearance: homogenous; color: yellowish; smell: specific
**Macroscopic characteristics after 60 days**	appearance: homogenous; color: yellowish smell: specific	appearance: homogenous; color: yellowish; smell: specific	appearance: homogenous; color: yellowish; smell: specific
**Initial pH**	6.70	6.60	6.40
**pH after 60 days**	6.70	6.50	6.30

**Table 5:** The determination coefficients, adjusted R^2^, and AIC_c_ values for different rheological models tested at 33 °C.

Gel/Rheological Model	Casson			Bingham			Ostwald-de Waele			Herschel-Bulkley		
	R^2^	Adj R^2^	AIC_c_	R^2^	Adj R^2^	AIC_c_	R^2^	Adj R^2^	AIC_c_	R^2^	Adj R^2^	AIC_c_
**Formula A**	0.9733	0.9715	33.29	0.8766	0.8684	59.29	0.9790	0.9776	29.16	0.9981	0.9978	−7.961
**Formula B**	0.9648	0.9624	50.01	0.8556	0.8460	74.01	0.9847	0.9837	35.85	0.9986	0.9984	−1.536
**Formula C**	0.9473	0.9437	77.29	0.8230	0.8112	97.87	0.9862	0.9853	54.51	0.9947	0.9939	41.870
**Formula D**	0.9605	0.9578	51.98	0.8462	0.8360	75.07	0.9832	0.9821	37.45	0.9969	0.9964	12.390
**Formula E**	0.9676	0.9654	47.99	0.8632	0.8541	72.47	0.9833	0.9822	36.73	0.9983	0.9981	1.090
**Formula F**	0.9672	0.9650	43.43	0.8603	0.8510	68.05	0.9840	0.9830	31.19	0.9990	0.9989	−12.980

**Table 6:** Herschel-Bulkey model flow parameters obtained through stationary shear analysis at 33 ± 0.1 °C for the designed carbopol gels.

Gel/ Flow Parameters	Yield Stress (Pa) (τ_0_—Pa)	Consistency Index (K—Pa·s^n^)	Flow Index (n)	Viscosity at 0.30 rpm (η_0.3_—Pa·s)	
				Initial	After 60 Days
**Formula A**	29.518	20.021	0.34	489.20	472.10
**Formula B**	48.333	34.938	0.29	822.90	710.20
**Formula C**	80.150	76.884	0.25	1553.00	1432.80
**Formula D**	51.397	36.334	0.28	871.90	760.30
**Formula E**	43.268	31.869	0.31	738.10	682.00
**Formula F**	41.732	28.915	0.30	698.80	536.40

**Table 7:** The influence of developed pharmaceutical formulations on selected pathogenic strains.

Strains	Samples											
	GB	B	E	CO	RO	TTO	UA	Complex	UA:CO	UA:RO	UA:TTO	DMSO
**Gram-positive bacteria**												
*E. faecalis* ATCC 29212	-	+	+	+++	+++	+++	+++	+++	+++	+++	+++	-
*S. aureus* ATCC 25923	-	+	+	+++	+	+	++	+++	++	+++	+++	-
**Gram-negative bacteria**												
*E. coli* ATCC 25922	+	+	+	++	++	++	++	++	++	++	++	-
*E. coli* 135	-	++	++	++	-	-	++	++	++	++	++	-
*E. coli* ESBL	-	++	++	++	++	++	++	++	++	++	++	-
*E. coli* 2425	+	++	+	++	++	++	++	++	++	++	++	-
*E. coli* 5075	-	++	++	+++	+++	+++	+++	+++	+++	+++	+++	-
*P. aeruginosa* ATCC 27853	-	+	++	+++	+++	+++	+++	+++	+++	+++	+++	-
**Yeasts**												
*C. albicans* ATCC 10231	-	++	++	+	+	++	++	++	++	++	++	-
*C. albicans* 5316	-	++	++	+++	+++	+++	+++	+++	+++	+++	+++	-
*C. albicans* 5319	-	+	+	+++	+++	+++	+	+	+++	+++	+++	+
*C. albicans* 5325	-	++	+	+++	+++	+++	+++	+++	+++	+++	+++	-
*C. albicans* 5328	-	++	++	++	++	++	++	++	++	+++	++	-
*C. krusei* 5299	-	++	++	++	++	++	++	++	++	++	++	-
*C. krusei* 5343	-	+	++	+	+	++	++	++	++	++	++	-
*C. glabrata* 5328	-	+	+	+	+	+	++	-	++	++	++	-
*C. parapsilosis* ATCC 22019	-	++	++	++	++	++	+++	+++	+++	+++	+++	-
*C. parapsilosis* 514	-	+	+	++	++	++	++	++	+++	+++	+++	+
*C. tropicalis* 5770	-	++	++	++	+	++	++	++	++	++	++	-

GB = gel base; B = formula B; E = formula E; CO = clove essential oil; RO = Rosemary essential oil; TTO = tea tree essential oil; UA = Usnic acid; Complex = Cyclodextrin complex with CO. (-)/pale green is represented by the absence of the inhibition; (+)/light green = GIZD ≤ 5 mm; (++)/green = GIZD between 5 and 10 mm; (+++)/forest green = GIZD between 10 and 18 mm. The significant impact of the formulas and their bioactive compounds on each microbial strain was statistically analyzed using one-way ANOVA and Tukey’s multiple comparisons test. The differences between groups were statistically significant (*p* < 0.05).

**Table 8:** Gels’ compositions.

Components	Formula A	Formula B	Formula C
**Carbopol 940**	0.8 g	1 g	1.2 g
**Glycerin**	5 g	5 g	5 g
**Triethanolamine**	q.s.	q.s.	q.s.
**Cyclodextrin complex with clove essential oil**	1 g	1 g	1 g
**Usnic acid**	0.5 g	0.5 g	0.5 g
**Tea tree essential oil**	1.5 g	1.5 g	1.5 g
**Rosemary essential oil**	1.5 g	1.5 g	1.5 g
**Purified water**	until 100 g	until 100 g	until 100 g

**Table 9:** Gels’ compositions.

Components	Formula D	Formula E	Formula F
**Carbopol 940**	1 g	1 g	1 g
**Glycerin**	5 g	5 g	5 g
**Triethanolamine**	q.s.	q.s.	q.s.
**Cyclodextrin complex with clove essential oil**	1 g	1 g	1 g
**Usnic acid**	0.5 g	0.5 g	0.5 g
**Tea tree essential oil**	1 g	2 g	2.5 g
**Rosemary essential oil**	1 g	2 g	2.5 g
**Purified water**	until 100 g	until 100 g	until 100 g

## Data Availability

The original contributions presented in the study are included in the article; further inquiries can be directed to the corresponding author.
